# A reusable neural network pipeline for unidirectional fiber segmentation

**DOI:** 10.1038/s41597-022-01119-6

**Published:** 2022-02-02

**Authors:** Alexandre Fioravante de Siqueira, Daniela M. Ushizima, Stéfan J. van der Walt

**Affiliations:** 1 Berkeley Institute for Data Science, University of California, Berkeley, 94720 USA; 2 Computational Research Division, Lawrence Berkeley National Laboratory, Berkeley, 94720 USA; 3 Bakar Computational Health Sciences Institute, University of California, San Francisco, 94143 USA

**Keywords:** Scientific data, Computational methods, Software

## Abstract

Fiber-reinforced ceramic-matrix composites are advanced, temperature resistant materials with applications in aerospace engineering. Their analysis involves the detection and separation of fibers, embedded in a fiber bed, from an imaged sample. Currently, this is mostly done using semi-supervised techniques. Here, we present an open, automated computational pipeline to detect fibers from a tomographically reconstructed X-ray volume. We apply our pipeline to a non-trivial dataset by Larson *et al*. To separate the fibers in these samples, we tested four different architectures of convolutional neural networks. When comparing our neural network approach to a semi-supervised one, we obtained Dice and Matthews coefficients reaching up to 98%, showing that these automated approaches can match human-supervised methods, in some cases separating fibers that human-curated algorithms could not find. The software written for this project is open source, released under a permissive license, and can be freely adapted and re-used in other domains.

## Introduction

Fiber-reinforced ceramic-matrix composites are advanced materials used in aerospace gas-turbine engines^1,2^ and nuclear fusion^3^, due to their resistance to temperatures 100–200 °C higher than alloys used in the same applications.

Larson *et al*. investigated new manufacturing processes for curing preceramic polymer into unidirectional fiber beds, studying the microstructure evolution during matrix impregnation with the aim of reinforcing ceramic-matrix composites^4,5^. They used X-ray computed tomography (CT) to characterize the three-dimensional microstructure of their composites non-destructively, studying their evolution *in-situ* while processing the materials at high temperatures^4^ and describing overall fiber bed properties and microstructures of unidirectional composites^5^. The X-ray CT images acquired from these fiber beds are available at Materials Data Facility^6^.

Larson *et al*.’s fiber beds have widths of approximately 1.5 *mm*, containing 5000–6200 fibers per stack. Each fiber has an average radius of 6.4 ± 0.9 *μm*, with diameters ranging from 13 to 20 pixels in the micrographs^5^. They present semi-supervised techniques to separate the fibers within the fiber beds; their segmentation is available for five samples^7^. We were curious to see whether their results could be improved using different techniques.

In this study we separate fibers in *ex-situ* X-ray CT fiber beds of nine samples from Larson *et al*. Our paper makes the following contributions:

- It annotates, explains, and expands Larson *et al*.’s dataset^7^ to facilitate reproducible research and benchmarking.
- It provides open source tools to analyze such datasets, so that researchers may compare their results with ours and one another’s.
- It shows that automated analysis can perform similarly to or better than human steered fiber segmentations.

The samples we used in this study correspond to two general states: wet — obtained after pressure removal — and cured. These samples were acquired using microtomographic instruments from the Advanced Light Source at Lawrence Berkeley National Laboratory operated in a low-flux, two-bunch mode^5^. We used their reconstructions obtained without phase retrieval; Larson *et al*. provide segmentations for five of these samples^7^, which we compare to our results.

To separate the fibers in these samples, we tested four different fully convolutional neural networks (CNN), algorithms from computer vision and deep learning. When comparing our neural network approach to Larson *et al*.’s results, we obtained Dice^8^ and Matthews^9^ coefficients greater than 92.28 ± 9.65%, reaching up to 98.42 ± 0.03%, showing that the network results are close to the human-supervised ones in these fiber beds, in some cases separating fibers that the algorithms created by Larson *et al*.^5^ could not find. All software and data generated in this study are available for download, along with instructions for their use. The code is open source, released under a permissive software license, and can be adapted easily for other domains.

## Results

**Fig. 1:**
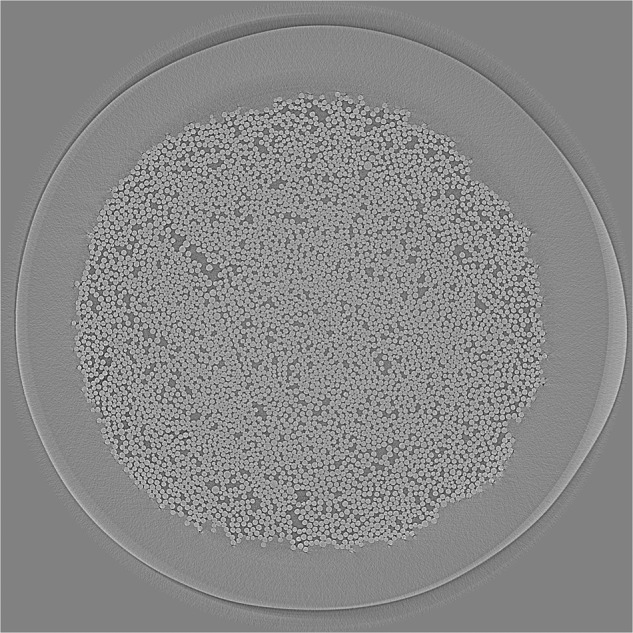
Slice number 1000 from the sample *“232p3 wet”*, provided in Larson *et al*.’s dataset^7^. The whole sample contains 2160 slices. This slice represents the structure of the samples we processed: they contain the fiber bed (large circular structure) and the fibers within it (small round elements).

Larson *et al*. provide segmentations for their fibers (Fig. 1) in five of the wet and cured samples, obtained using the following pipeline^5^:

1. Fiber detection using the circular Hough transform^10,11^;
2. Correction of improperly identified pixels using filters based on connected region size and pixel value, and by comparisons using ten slices above and below the slice of interest;
3. Separation of fibers using the watershed algorithm^12^.

**Fig. 2:**
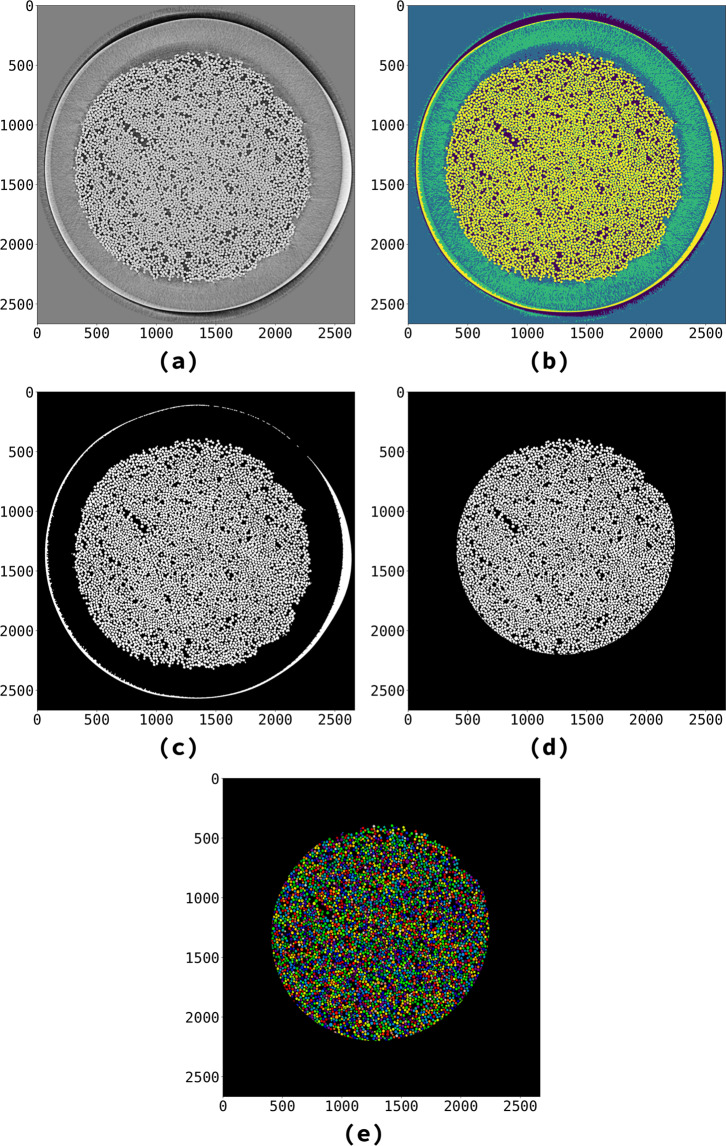
Rendering fibers detected in the limited region of interest by the classic pipeline. We illustrate the classic image processing pipeline using Fig. 1 as the input image. This solution had limitations when processing fibers on the edges of fiber beds. **(a)** Histogram equalization and TV Chambolle’s filtering (parameter: ﻿weight = 0.3). **(b)** Multi Otsu’s resulting regions (parameter: classes = 4). Fibers are located within the fourth region (in yellow). **(c)** Binary image obtained considering region four in (**b**) as the region of interest, and the remaining regions as the background. (**d**) the processed region from (**c**), as shown in Fig. 1. (**e**) Regions resulting from the application of WUSEM on the region shown in (**d**) (parameters: initial_radius = 0, delta_radius = 2, watershed_line = True). Colormaps: (**a**,**c**,**d**) gray, (**b**) viridis, (**e**) nipy_spectral.

Their paper gives a high-level overview of these steps, but provides no details on parameters used, nor the source code for computing their segmentation. We tried different approaches to reproduce their results, focusing on separating the fibers in the fiber bed samples. Our first approach was to create a classic, unsupervised image processing pipeline. We used histogram equalization^13^, Chambolle’s total variation denoising^14,15^, multi-Otsu threshold^16,17^, and the WUSEM algorithm^18^ to separate each single fiber. The result is a labeled image containing the separated fibers (Fig. 2). The pipeline had limitations when processing fibers on the edges of fiber beds, where its labels differed from those produced by Larson *et al*. Restricting the segmentation region to the center of beds gives satisfactory results (Fig. 2(e)), but reduces the total number of detected fibers.

To obtain more robust results, we evaluated four fully convolutional neural network architectures: Tiramisu^19^ and U-Net^20^, as well as their three-dimensional counterparts, 3D Tiramisu and 3D U-Net^21^. We also investigated whether three-dimensional networks generate better segmentation results, leveraging the structure of the material.

### Fully convolutional neural networks (CNN) for fiber detection

We implemented four architectures of fully convolutional neural networks (CNNs) — Tiramisu, U-Net, 3D Tiramisu, and 3D U-Net — to reproduce the results provided by Larson *et al*. Labeled data, in our case, consists of fibers within fiber beds. To train the neural networks to recognize these fibers, we used slices from two different samples: *“232p3 wet”* and *“232p3 cured”*, registered according to the wet sample. Larson *et al*. provided the fiber segmentation for these samples^7^, which we used as labels in the training. The training and validation datasets contained 250 and 50 images from each sample, respectively, in a total of 600 images. Each image from the original samples have width and height size of 2560 × 2560 pixels.

**Fig. 3:**
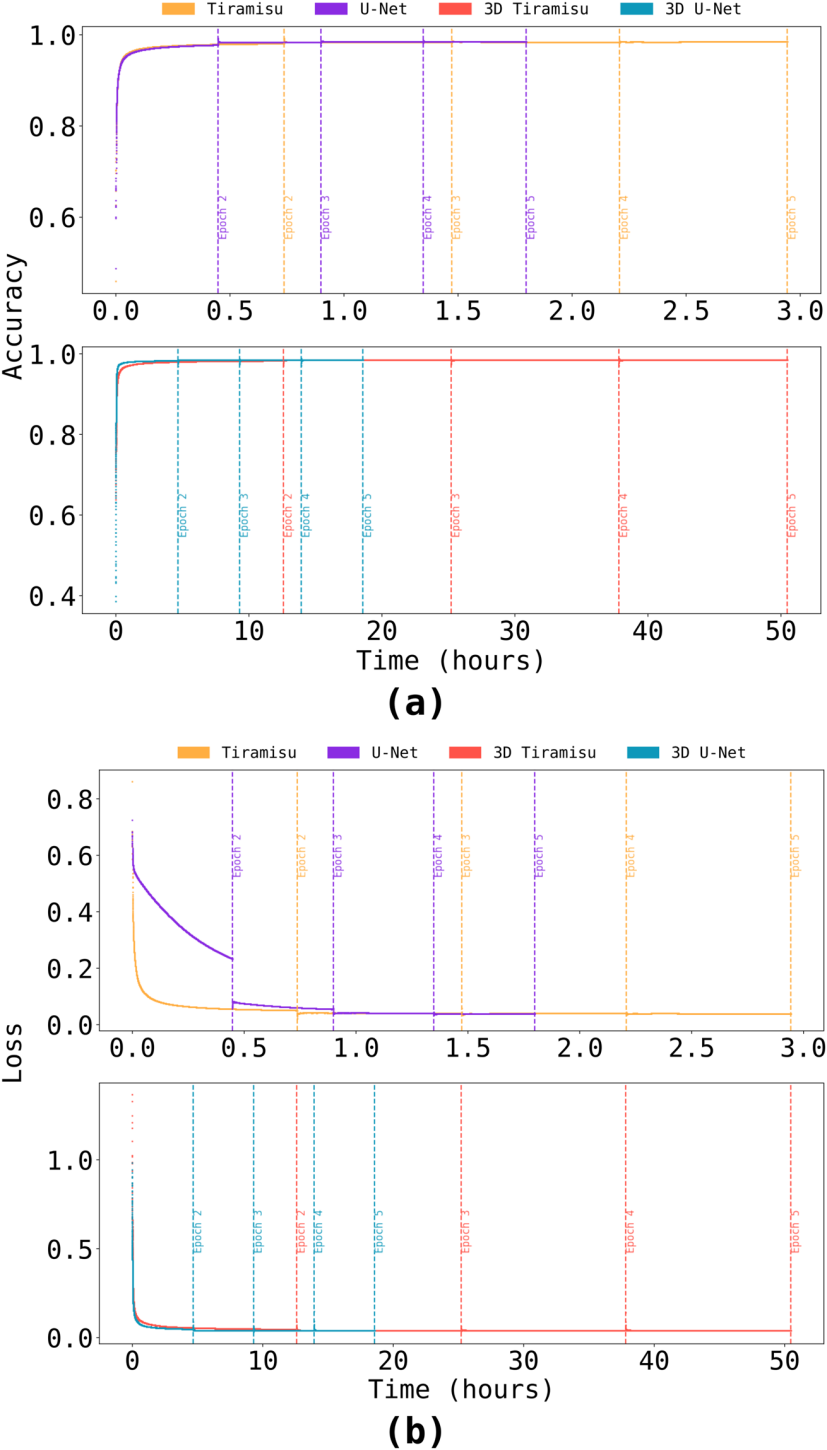
Accuracy **(a)** and loss **(b)** through time for each training epoch. We attribute the subtle loss increase or accuracy decrease on the start of each epoch to the data augmentation process.

For all networks, we used a learning rate of 1^−4^, and binary cross entropy^22^ as the loss function. During training, the networks reached accuracy higher than 0.9 and loss lower than 0.1 on the first epoch. Two-dimensional U-Net is the exception, presenting loss of 0.23 at the end of the first epoch. Despite that, 2D U-Net reaches the lowest loss between the four architectures at the end of its training. 2D U-Net is also the fastest network to finish its training (7 h, 43 min), followed by Tiramisu (13 h, 10 min), 3D U-Net (24 h, 16 min) and 3D Tiramisu (95 h, 49 min, Fig. 3).

**Fig. 4:**
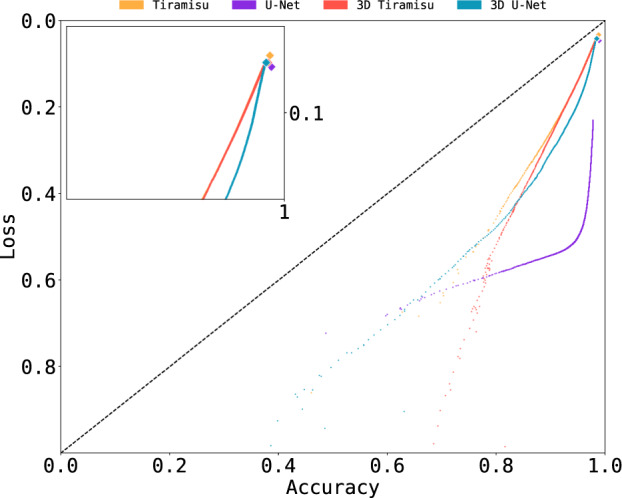
Accuracy vs. loss on the first epoch. Accuracy surpasses 0.9 and loss is lower than 0.1 for all networks during the first epoch, except for 2D U-Net (loss of 0.23). Validation accuracy and validation loss on the first epoch are represented by diamonds.

Examining convergence behavior on the first epoch, the 2D U-Net does not progress as smoothly as the other networks (Fig. 4). However, this does not impair U-Net’s accuracy (0.977 after one epoch). Accuracy and loss for the validation dataset also improve significantly: Tiramisu had validation loss vs. validation accuracy ratio of 0.034 while U-Net had 0.048, and both 3D architectures had ratios of 0.043. The large size of the training set and the similarities between slices in the input data are responsible for these high accuracies and low losses.

We used the trained networks to predict fiber labelings for twelve different datasets in total. These datasets were made available by Larson *et al*.^7^, and we keep the same file identifiers for fast cross-reference:

- **“232p1”**: wet
- **“232p3”**: wet, cured, cured registered
- **“235p1”**: wet
- **“235p4”**: wet, cured, cured registered
- **“244p1”**: wet, cured, cured registered
- **“245p1”**: wet

Here, the first three numeric characters correspond to a sample, and the last character correspond to different extrinsic factors, e.g. deformation. Despite being samples from similar materials, the reconstructed files presented several differences, for example regarding amount of ringing artifacts, intensity variation, noise, therefore they are considered as different samples in this paper.

**Fig. 5:**
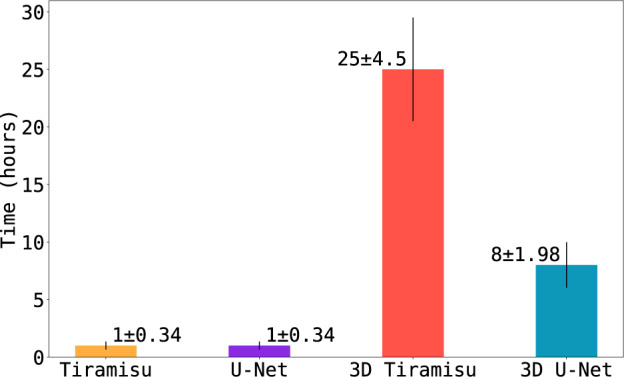
Mean and standard deviation of prediction times for each sample. As with processing, during training 2D U-Net and 2D Tiramisu were the fastest architectures to process a sample in one hour, on average. 3D Tiramisu, being the slowest, takes on average more than a day to process one sample.

We calculated the average prediction time for each sample (Fig. 5). As with the training time results, 2D U-Net and 2D Tiramisu are the fastest architectures to process a sample, while 3D Tiramisu is the slowest.

### Evaluation of our results and comparison with Larson *et al*. (2019)

After processing all samples, we compared our predictions with the results that Larson *et al*. made available on their dataset^7^. They provided segmentations for five datasets from the twelve we processed: *“232p1 wet”*, *“232p3 cured”*, *“232p3 wet”*, *“244p1 cured”*, *“244p1 wet”*.

**Fig. 6:**
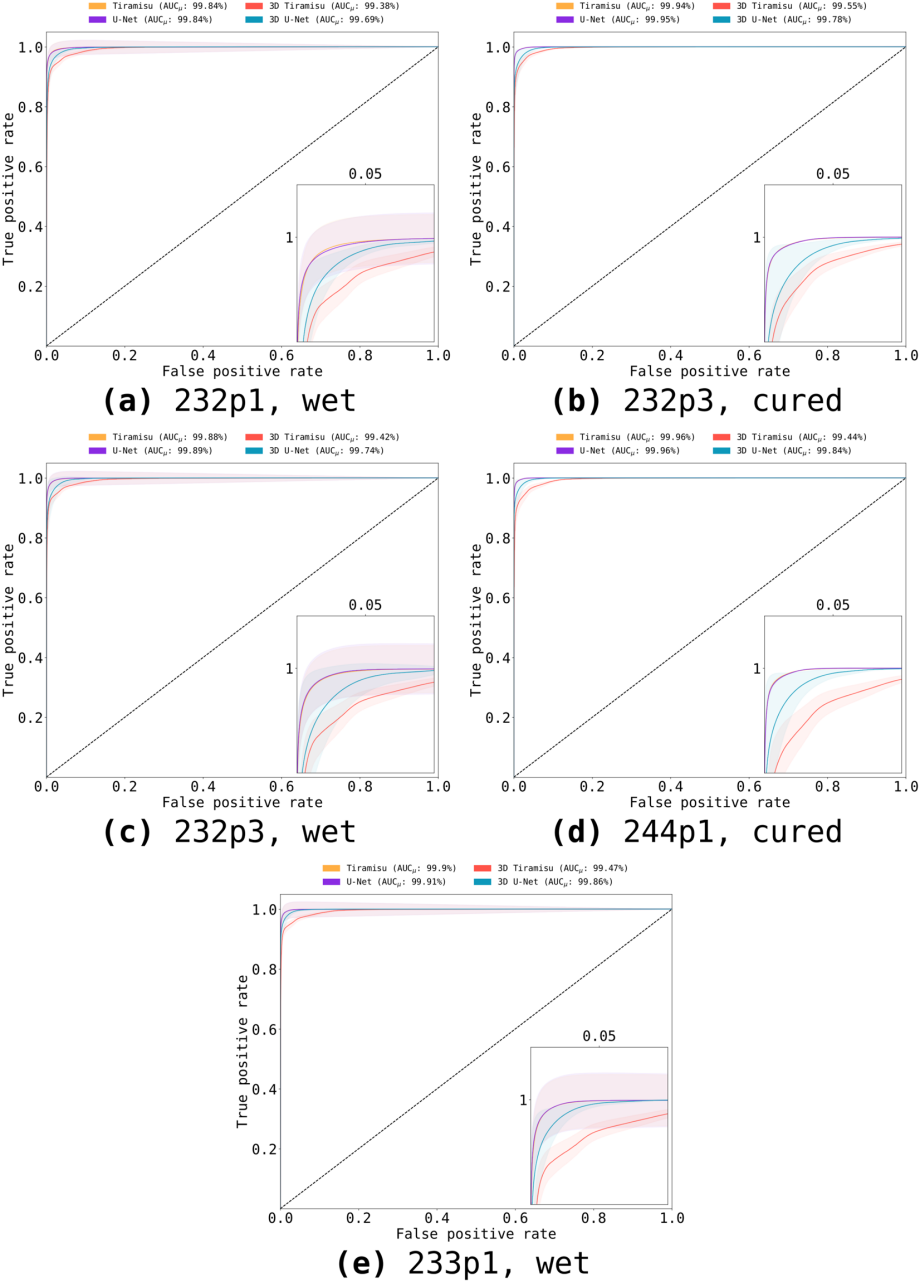
Receiver operating characteristic (ROC) and area under curve (AUC) obtained from the comparison between prediction and gold standard. We consider Larson *et al*.’s segmentation^7^ as the gold standard in this case. ROC curves were calculated for all slices in each dataset; their mean areas and standard deviation intervals are presented. AUC is larger than 98% in all comparisons.

First, we compared our predictions to their results using receiver operating characteristic (ROC) curves and the area under curve (AUC, Fig. 6). AUC is larger than 98% for all comparisons; therefore, our predictions are accurate when compared with the semi-supervised method suggested by Larson *et al*.^5^. The 2D versions of U-Net and Tiramisu have similar results, performing better than 3D U-Net and 3D Tiramisu.

We also examined the binary versions of our predictions and compared them with Larson *et al*.’s results. For each slice or cube from the dataset, we used a hard threshold of 0.5; values above that are considered as fibers, while values below that are treated as background. We used Dice^8^ and Matthews^9^ correlation coefficients for our comparison (Table ﻿1). The comparison using U-Net yields the highest Dice and Matthews coefficients for three of five datasets. Tiramisu had the highest Dice/Matthews coefficients for the *“244p1 cured”* dataset, and both networks have similar results for *“232p1 wet”*. 3D Tiramisu had the lowest Dice and Matthews coefficients in our comparison.

## Discussion

The analysis of ceramic matrix composites (CMC) depends on the detection of its fibers. Semi-supervised algorithms, such as the one presented by Larson *et al*.^5^, can perform that task satisfactorily. The description of that specific algorithm, however, lacks information on parameters necessary for replication. It also includes steps that involve manual curation. As such, it was not possible for us to reimplement it fully.

Convolutional neural networks are being used successfully in the segmentation of different two- and three-dimensional scientific data^23–28^, including microtomographies. For example, fully convolutional neural networks were used to generate 3D tau inclusion density maps^29^, to segment the tidemark on osteochondral samples^30^, and 3D models of structures of temporal-bone anatomy^31^.

Researchers have been studying fiber-analysis detection for a while, using a variety of tools. Approaches include tracking, statistical methods, and classical image processing^32–39^. To the best of our knowledge, there are two different deep learning approaches applied to this problem:

- Yu *et al*.^40^ use an unsupervised learning approach based on Faster R-CNN^41^ and a Kalman filter based tracking. They compare their results with Zhou *et al*.^36^, reaching a Dice coefficient of up to 99%.
- Miramontes *et al*.^42^ reach an average accuracy of 93.75% using a 2D LeNet-5 CNN^43^ to detect fibers in a specific sample.

Our study builds upon previous work by using similar material samples, but it expands tests to many more samples and it includes the implementation and training of four architectures: 2D U-Net, 2D Tiramisu, 3D U-Net, and 3D Tiramisu, used to process twelve large datasets (≈140 GB total), and comparing our results with the gold standard labeling provided by Larson *et al*.^7^ for five of them. We used ROC curves and their area under curve (AUC) to ensure the quality of our predictions, obtaining AUC larger than 98% (Fig. 6). Also, Dice and Matthews coefficients were used to compare our results with Larson *et al*.’s solutions (Table 1), reaching coefficients of up to 98.42 ± 0.03%.

**Table 1:** Dice and Matthews coefficients for each sample, obtained from the comparison of our neural network results and data from Larson *et al*.^7^.

Sample	Tiramisu		U-Net		3D Tiramisu		3D U-Net	
	Dice	Matthews	Dice	Matthews	Dice	Matthews	Dice	Matthews
**232p1, wet**	97.58 ± 2.29%	96.55 ± 2.93%	97.58 ± 2.20%	96.60 ± 2.13%	94.54 ± 6.73%	92.28 ± 9.65%	95.59 ± 0.74%	93.71 ± 1.03%
**232p3, cured**	98.21 ± 0.04%	97.47 ± 0.06%	98.26 ± 0.04%	97.53 ± 0.06%	95.25 ± 6.36%	93.39 ± 8.88%	95.90 ± 1.00%	94.21 ± 1.30%
**232p3, wet**	97.79 ± 2.15%	96.87 ± 2.70%	97.85 ± 2.12%	96.98 ± 1.99%	94.86 ± 6.90%	92.76 ± 9.87%	95.68 ± 1.97%	93.92 ± 2.36%
**244p1, cured**	98.42 ± 0.03%	97.83 ± 0.05%	98.38 ± 0.04%	97.78 ± 0.05%	94.55 ± 7.74%	92.67 ± 10.54%	96.30 ± 1.25%	94.97 ± 1.54%
**244p1, wet**	98.08 ± 2.53%	97.39 ± 3.15%	98.10 ± 2.39%	97.43 ± 2.23%	94.81 ± 7.81%	92.97 ± 10.71%	96.67 ± 1.00%	95.45 ± 1.31%

U-Net yields the highest Dice and Matthews coefficients for three of five samples. Tiramisu had highest Dice/Matthews coefficients for one of the datasets. 3D Tiramisu had the lowest Dice and Matthews coefficients.

**Fig. 7:**
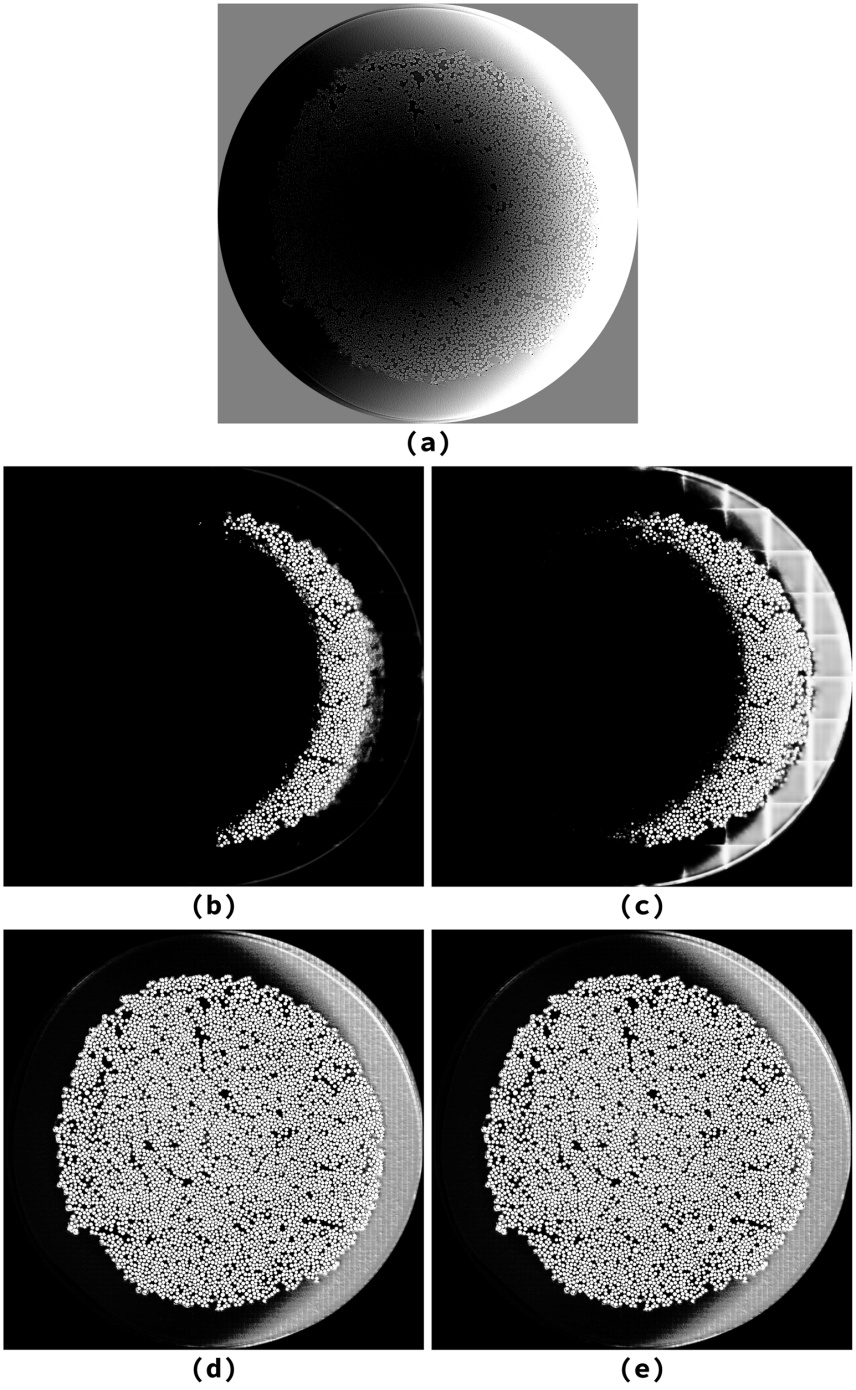
A defective slice on the sample *“232p3 wet”* and the segmentation produced by each architecture. Segmentations computed by 2D architectures are impaired by defects in the input image, while 3D architectures leverage the sample structure to achieve better results. **(a)** Original defective image, **(b)** U-Net prediction, **(c)** 3D U-Net prediction, **(d)** Tiramisu prediction, **(e)** 3D Tiramisu prediction.

When processing a defective slice (a slice with severe artifacts), the 3D architectures perform better than the 2D ones since they are able to leverage information about the structure of the material (Fig. 7).

Based on the research presented, we recommend using the 2D U-Net to process microtomographies of CMC fibers. Both 2D networks lead to similar accuracy and loss values in our comparisons (Table 1); however, U-Nets converge more rapidly and are therefore computationally cheaper to train than Tiramisu. The 3D architectures, while performing better on defective samples (Fig. 7), do not generally achieve better results than the 2D architectures. In fact, the 3D architectures require more training to achieve comparable accuracy (Fig. 3) and are slower to predict (Fig. 5), therefore requiring considerable additional computation for marginal gains.

**Fig. 8:**
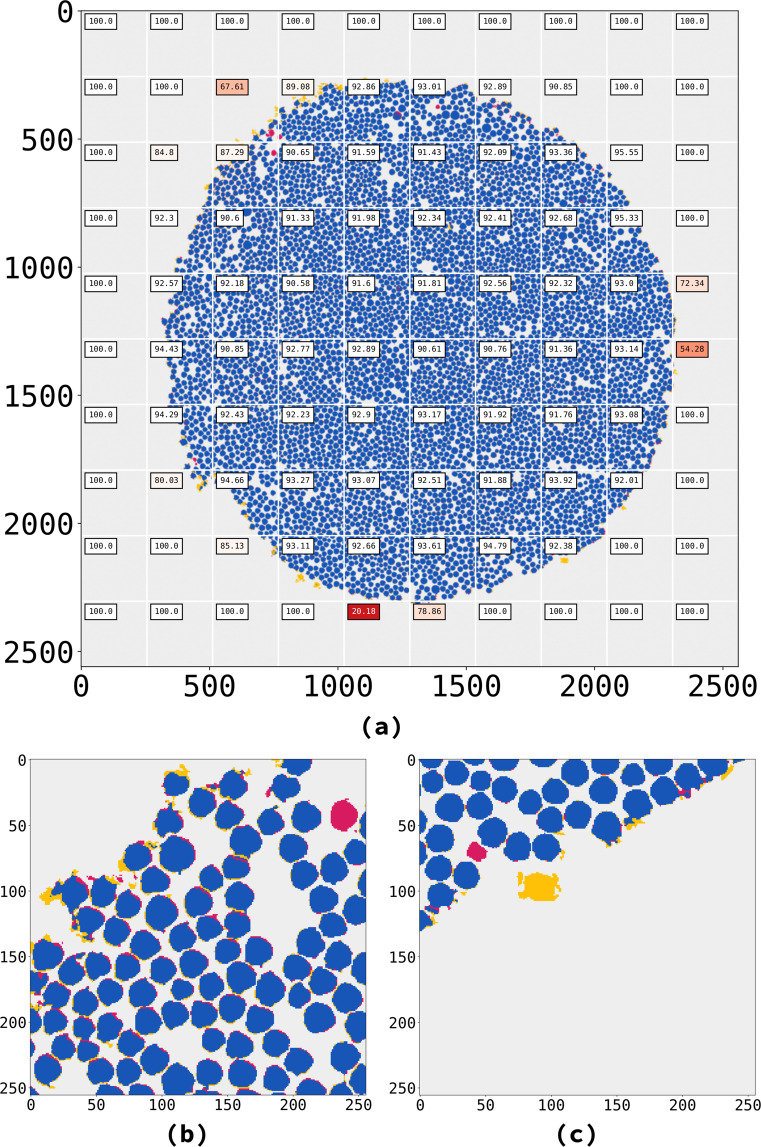
**(a)** Visual comparison between 2D U-Net and Larson *et al*.’s results for sample *“232p3 wet”*. We divided the slices into 100 tiles, and compared each tile from our U-Net prediction to Larson *et al*.’s corresponding labels. The tiles presented here are the ones that return the lowest Matthews comparison coefficients. Labels present the Matthews coefficient for each tile. (**b**,**c**) tiles showing fibers found only by U-Net (in red), while some well-defined structures close to the borders are found only by Larson *et al*. (in yellow). Tile size: 256 × 256. Colors set according to the comparison. Blue: true positives; red: false positives; yellow: false negatives; gray: true negatives.

Our CNN architectures perform at the level of human-curated accuracy — i.e., Larson *et al*.’s semi-supervised approach —, sometimes even surpassing it. For instance, the 2D U-Net identified fibers that the Larson *et al*. algorithm did not find (Fig. 8).

**Fig. 9:**
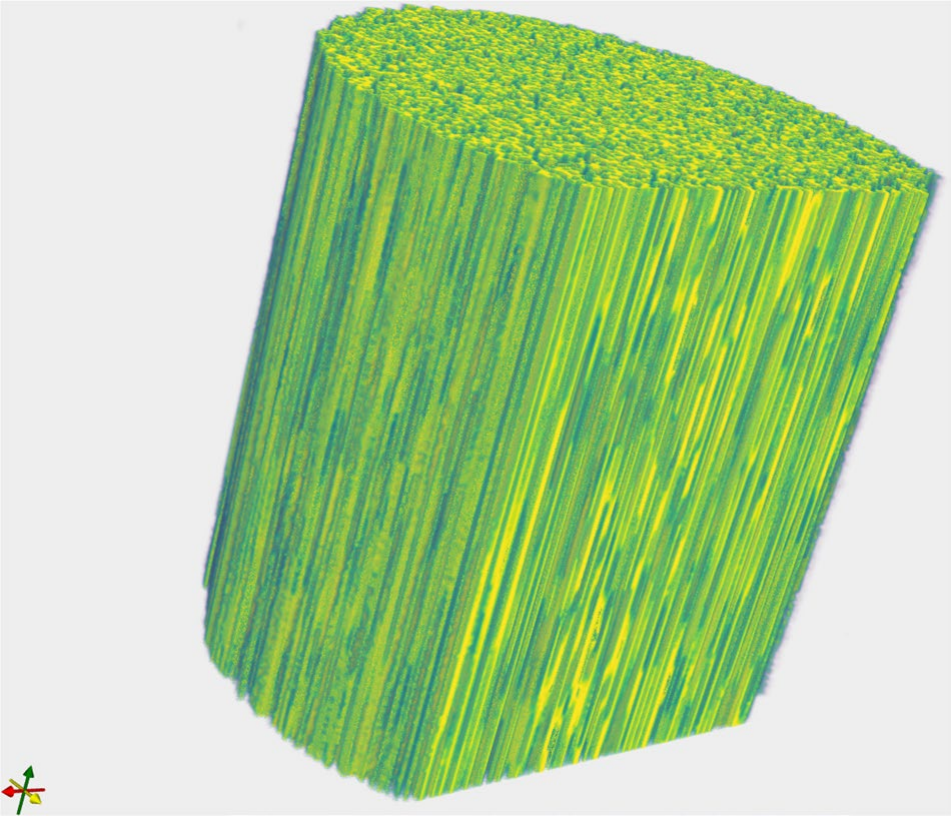
Fibers on the sample *“232p3 wet”* processed using the U-Net architecture. As seen in the longitudinal cut, this pipeline identifies fibers across the sample height despite the absence of tracking.

Using labels predicted by the U-Net architecture, we render a three-dimensional visualization of the fibers (Fig. 9). Despite the absence of tracking, the U-Net segmentation clearly outlines fibers across the stack.

In this paper, we presented neural networks for analyzing microtomographies of CMC fibers in fiber beds. The data used is publicly available^7^ and was acquired in a real materials design experiment. Results are comparable to human-curated segmentations; yet, the networks can predict fiber locations in large stacks of microtomographies without any human intervention. Despite the encouraging results achieved in this study, there is room for improvement. For example, the training time of especially the 3D networks turned out to be prohibitive in performing a full hyperparameter sweep. A search for optimal parameters of all networks used could be implemented in a future study. We also aim to investigate whether an ensemble of networks will perform better. We would also like to explore how to best adjust thresholds at the last layer of the network. Here, we maintained a hard threshold of 0.5 that suited the sigmoid on the last layer of the implemented CNNs, but one could, e.g., use conditional random field networks instead.

## Methods

### Fully convolutional neural networks

We implemented four architectures — two dimensional U-Net^20^ and Tiramisu^19^, and their three-dimensional versions — to attempt improving on the results provided by Larson *et al*. We used supervised algorithms: they rely on labeled data to learn what are the regions of interest — in our case, fibers within microtomographies of fiber beds.

All CNN algorithms were implemented using TensorFlow^44^ and Keras^45^ on a computer with two Intel Xeon Gold processors 6134 and two Nvidia GeForce RTX 2080 graphical processing units. Each GPU has 10 GB of RAM.

To train the neural networks in recognizing fibers, we used slices from two different samples: *“232p3 wet”* and *“232p3 cured”*, registered according to the wet sample. Larson *et al*. provided the fiber segmentation for these samples, which we used as labels in the training. The training and validation procedures processed 350 and 149 images from each sample, respectively; a total of 998 images. Each image from the original samples have width and height size of 2560 × 2560 pixels.

To feed the two-dimensional networks, we padded the images with 16 pixels, of value zero, in each dimension. Then, each image was cut into tiles of size 288 × 288, each 256 pixels, creating an overlap of 32 pixels. These overlapping regions, which are removed after processing, avoid artifacts on the borders of processed tiles. Therefore, each input slice generated 100 images with 288 × 288 pixels, in a total of 50,000 images for the training set, and 10,000 for the validation set.

We needed to pre-process the training images differently to train the three-dimensional networks. We loaded the entire samples, each with size 2160 × 2560 × 2560, and padded their dimensions with 16 pixels of value zero. Then, we cut slices of size 64 × 64 × 64 voxels, each 32 pixels. Hence, the training and validation sets for the three-dimensional networks have 96,000 and 19,200 cubes, respectively.

We implemented data augmentation, aiming for a network capable of processing samples with varying characteristics. We augmented the images on the training sets using rotations, horizontal and vertical flips, width and height shifts, zoom, and shear transforms. For that, we used Keras embedded tools within the ImageDataGenerator module to augment images for the two-dimensional networks. Since Keras’s ImageDataGenerator is not able to process three-dimensional input so far, we adapted the ImageDataGenerator module. The adapted version we used in this study is named ChunkDataGenerator, and is provided at the repository presented in the section Code Availability, along with the software produced in this study.

To reduce the possibility of overfitting, we implemented dropout regularization^46^. We followed the suggestions in the original papers for U-Net architectures: 2D U-Net received a dropout rate of 50% in the last analysis layer and in the bottleneck, while 3D U-Net^21^ did not receive any dropout. The Tiramisu structures received a dropout rate of 20%, as suggested by Jégou *et al*.^19^.

#### Hyperparameters

To better compare the networks, we maintain the same training hyperparameters when possible. Ideally, we would conduct a hyperparameter sweep — a search for the optimal hyperparameters for each network —, but training time turned out to be prohibitive, especially for the three-dimensional networks. Due to the large amount of training data and the similarities between training samples (2D tiles or 3D cubes), we decided to train all architectures for five epochs. The 2D architectures were trained with batches of four images, while the batches for 3D architectures had two cubes each. The learning rate used was 1^−4^, and the loss function used was binary cross entropy^22^. We followed advice from the original papers with regards to optimization algorithms: we used the Adam optimizer^47^ for U-Net architectures, and RMSProp^48^ for Tiramisu. We implemented batch normalization^49^ in all architectures, including the 2D U-Net. While Ronneberger *et al*.^20^ does not discuss batch normalization explicitly, it has been shown to improve convergence^49^.

### Evaluation

We used Dice^8^ and Matthews^9^ correlation coefficients (Eqs. 1, 2) to evaluate our results, assuming that the fiber detections from Larson *et al*.^7^ are a reasonable gold standard.

1 $$Dice=\frac{2\times TP}{2\times TP+FP+{\rm{FN}}}$$

2 $$Matthews=\frac{TP\times TN-FP\times FN}{\sqrt{(TP+FN)(TP+FP)(TN+FN)(TN+FP)}}$$

Dice and Matthews coefficients receive true positive (TP), false positive (FP), true negative (TN), and false negative (FN) pixels, which are determined as:

- **TP:** pixels correctly labeled as being part of a fiber.
- **FP:** pixels incorrectly labeled as being part of a fiber.
- **TN:** pixels correctly labeled as background.
- **FN:** pixels incorrectly labeled as background.

TP, FP, TN, and FN are obtained when the prediction data is compared with the gold standard.

### Dataset

The dataset accompanying Larson *et al*.^5^ includes raw images, segmentation results, and a brief description of segmentation tools — the Hough transform, mathematical morphology, and statistical filters. To reproduce their work fully would have required further information, including metadata, parameters used and, ideally, code for analysis. To aid reproducing segmentation results, we contribute a set of twelve processed fiber beds, based on the Larson *et al*. data. We also include the weights for each neural network architecture we implemented and trained. These weights can be used to process fibers of similar structure in other datasets.

### Visualization

Imaging CMC specimens at high-resolution, such as the Larson *et al*. samples^7^, leads to large datasets — for example, each stack we used in this paper occupies around 14 GB after reconstruction, with the following exceptions: the registered versions of cured samples 232p3, 235p4 and 244p1, at 11 GB each, and the sample 232p3 wet at around 6 GB.

Often, specialists need software to visualize results during data collection. Yet, it can be challenging to produce meaningful figures without advanced image analysis and/or computational platforms with generous amounts of memory. We wanted to show that interactive exploration of large datasets is viable on a modest laptop computer. We therefore used matplotlib^50^ and ITK^51^ (Fig. 9) to generate all figures in this paper, using a standard laptop with 16 GB of RAM. This means that a scientist could use, e.g., Jupyter Notebooks^52^ to do quick, interactive probing of specimens during beamtime.

## Data Availability

This study uses neural networks to process fibers in fiber beds, using Larson *et al*. datasets^7^. To be able to reproduce our study, it is necessary to download that data. We used twelve different datasets in total. We keep the same file identifiers Larson *et al*. used in their study, for fast cross-reference: • **“232p1”:**    — *wet:* folder data/Recons/Bunch2WoPR/rec20160324_055424_232p1_wet_1cm_cont_4097im_1500ms_17keV_13_a.h5 • **“232p3”:**    — *wet:* folder data/Recons/Bunch2WoPR/rec20160318_191511_232p3_2cm_cont__4097im_1500ms_ML17keV_6.h5 — *cured:* folder data/Recons/Bunch2WoPR/rec20160323_093947_232p3_cured_1p5cm_cont_4097im_1500ms_17keV_10.h5 — *cured registered:* folder data/Seg/Bunch2/rec20160323_093947_232p3_cured_1p5cm_cont_4097im_1500ms_17keV_10.h5/Registered/Bunch2WoPR • **“235p1”:**    — *wet:* folder data/Recons/Bunch2WoPR/rec20160324_123639_235p1_wet_0p7cm_cont_4097im_1500ms_17keV_14.h5 • **“235p4”:**    — *wet:* folder data/Recons/Bunch2WoPR/rec20160326_175540_235p4_wet_1p15cm_cont_4097im_1500ex_17keV_20.h5 — *cured:* folder data/Recons/Bunch2WoPR/rec20160327_003824_235p4_cured_1p15cm_cont_4097im_1500ex_17keV_22.h5 — *cured registered:* folder data/Seg/Bunch2/rec20160327_003824_235p4_cured_1p15cm_cont_4097im_1500ex_17keV_22.h5/Registered/Bunch2WoPR • **“244p1”:**    — *wet:* folder data/Recons/Bunch2WoPR/rec20160318_223946_244p1_1p5cm_cont__4097im_1500ms_ML17keV_7.h5 — *cured:* folder data/Recons/Bunch2WoPR/rec20160320_160251_244p1_1p5cm_cont_4097im_1500ms_ML17keV_9.h5 — *cured registered:* folder data/Seg/Bunch2/rec20160320_160251_244p1_1p5cm_cont_4097im_1500ms_ML17keV_9.h5/Registered/Bunch2WoPR • **“245p1”:**    — *wet:* folder rec20160327_160624_245p1_wet_1cm_cont_4097im_1500ex_17keV_23.h5 The first three numeric characters correspond to a sample, and the last character correspond to different extrinsic factors, e.g. deformation. Despite being samples from similar materials, the reconstructed files presented several differences: different amount of ringing artifacts, intensity variation, noise, etc. The data generated in this study is available in Dryad^53^, under a CC0 license. CC0 dedicates the work to the public domain, to the extent allowed by law.

## Code availability

The software produced throughout this study is available at https://github.com/alexdesiqueira/fcn_microct/ under the BSD-3 license.

## References

[1] Zok FW (2016). Ceramic-matrix composites enable revolutionary gains in turbine engine efficiency. American Ceramic Society Bulletin.

[2] Padture NP (2016). Advanced structural ceramics in aerospace propulsion. Nature Materials.

[3] Koyanagi T (2018). Recent progress in the development of sic composites for nuclear fusion applications. Journal of Nuclear Materials.

[4] Larson NM, Zok FW (2018). *In-situ* 3d visualization of composite microstructure during polymer-to-ceramic conversion. Acta Materialia.

[5] Larson NM, Cuellar C, Zok FW (2019). X-ray computed tomography of microstructure evolution during matrix impregnation and curing in unidirectional fiber beds. Composites Part A: Applied Science and Manufacturing.

[6] Blaiszik B (2016). The materials data facility: Data services to advance materials science research. JOM.

[7] Larson NM, Zok FW (2019). Materials Data Facility.

[8] Dice LR (1945). Measures of the amount of ecologic association between species. Ecology.

[9] Matthews BW (1975). Comparison of the predicted and observed secondary structure of t4 phage lysozyme. Biochimica et Biophysica Acta (BBA) - Protein Structure.

[10] Yuen, H. K., Princen, J., Dlingworth, J. & Kittler, J. A Comparative Study of Hough Transform Methods for Circle Finding. In *Procedings of the Alvey Vision Conference 1989*, 29.1–29.6, 10.5244/C.3.29 (Alvey Vision Club, Reading, 1989).

[11] Atherton T, Kerbyson D (1999). Size invariant circle detection. Image and Vision Computing.

[12] Meyer F (1994). Topographic distance and watershed lines. Signal Processing.

[13] Woods R, Gonzalez R (1981). Real-time digital image enhancement. Proceedings of the IEEE.

[14] Rudin LI, Osher S, Fatemi E (1992). Nonlinear total variation based noise removal algorithms. Physica D: Nonlinear Phenomena.

[15] Chambolle A (2004). An algorithm for total variation minimization and applications. Journal of Mathematical Imaging and Vision.

[16] Otsu N (1979). A threshold selection method from gray-level histograms. IEEE Transactions on Systems, Man and Cybernetics.

[17] Liao P-S, Chen T-S, Chung P-C (2001). A fast algorithm for multilevel thresholding. Journal of Information Science and Engineering.

[18] de Siqueira AF, Nakasuga WM, Guedes S, Ratschbacher L (2019). Segmentation of nearly isotropic overlapped tracks in photomicrographs using successive erosions as watershed markers. Microscopy Research and Technique.

[19] Jégou, S., Drozdzal, M., Vazquez, D., Romero, A. & Bengio, Y. The one hundred layers tiramisu: Fully convolutional densenets for semantic segmentation. *arXiv:1611.09326 [cs]* ArXiv: 1611.09326 (2017).

[20] Ronneberger, O., Fischer, P. & Brox, T. U-Net: Convolutional Networks for Biomedical Image Segmentation. In Navab, N., Hornegger, J., Wells, W. M. & Frangi, A. F. (eds.) *Medical Image Computing and Computer-Assisted Intervention* – *MICCAI 2015*, Lecture Notes in Computer Science, 234–241 (Springer International Publishing, 2015).

[21] çiçek, Ö., Abdulkadir, A., Lienkamp, S. S., Brox, T. & Ronneberger, O. 3d u-net: Learning dense volumetric segmentation from sparse annotation. *arXiv:1606.06650 [cs]* ArXiv: 1606.06650 (2016).

[22] Zhang, Z. & Sabuncu, M. R. Generalized cross entropy loss for training deep neural networks with noisy labels. In *Proceedings of the 32nd International Conference on Neural Information Processing Systems*, NIPS’18, 8792–8802 (Curran Associates Inc., 2018). PMC11747755 39839708

[23] Banerjee S (2020). Semantic segmentation of microscopic neuroanatomical data by combining topological priors with encoder–decoder deep networks. Nature Machine Intelligence.

[24] Tokuoka Y (2020). 3d convolutional neural networks-based segmentation to acquire quantitative criteria of the nucleus during mouse embryogenesis. npj Systems Biology and Applications.

[25] Horwath JP, Zakharov DN, Mégret R, Stach EA (2020). Understanding important features of deep learning models for segmentation of high-resolution transmission electron microscopy images. npj Computational Materials.

[26] Ma B (2020). Data augmentation in microscopic images for material data mining. npj Computational Materials.

[27] Saito Y (2019). Deep-learning-based quality filtering of mechanically exfoliated 2d crystals. npj Computational Materials.

[28] Li W, Field KG, Morgan D (2018). Automated defect analysis in electron microscopic images. npj Computational Materials.

[29] Alegro, M. *et al*. Deep learning for alzheimer’s disease: Mapping large-scale histological tau protein for neuroimaging biomarker validation. *bioRxiv* 698902, 10.1101/698902 (2020). 10.1016/j.neuroimage.2021.118790 PMC8983026 34933123

[30] Tiulpin, A., Finnilä, M., Lehenkari, P., Nieminen, H. J. & Saarakkala, S. Deep-learning for tidemark segmentation in human osteochondral tissues imaged with micro-computed tomography. In Blanc-Talon, J., Delmas, P., Philips, W., Popescu, D. & Scheunders, P. (eds.) *Advanced Concepts for Intelligent Vision Systems*, Lecture Notes in Computer Science, 131–138, 10.1007/978-3-030-40605-9_12 (Springer International Publishing, 2020).

[31] Nikan, S., Agrawal, S. K. & Ladak, H. M. Fully automated segmentation of the temporal bone from micro-ct using deep learning. In *Medical Imaging 2020: Biomedical Applications in Molecular, Structural, and Functional Imaging*, **11317**, 113171U, 10.1117/12.2549609 (International Society for Optics and Photonics, 2020).

[32] Czabaj MW, Riccio ML, Whitacre WW (2014). Numerical reconstruction of graphite/epoxy composite microstructure based on sub-micron resolution x-ray computed tomography. Composites Science and Technology.

[33] Bricker, S., Simmons, J. P., Przybyla, C. & Hardie, R. Anomaly detection of microstructural defects in continuous fiber reinforced composites. In Bouman, C. A. & Sauer, K. D. (eds.) *Annals of the SPIE/IS&T Electronic Imaging*, 94010A, 10.1117/12.2079679 (2015).

[34] Sencu RM (2016). Generation of micro-scale finite element models from synchrotron x-ray ct images for multidirectional carbon fibre reinforced composites. Composites Part A: Applied Science and Manufacturing.

[35] Ushizima DM (2016). Ideal: Images across domains, experiments, algorithms and learning. JOM.

[36] Zhou Y, Yu H, Simmons J, Przybyla CP, Wang S (2016). Large-scale fiber tracking through sparsely sampled image sequences of composite materials. IEEE Transactions on Image Processing.

[37] Emerson MJ, Jespersen KM, Dahl AB, Conradsen K, Mikkelsen LP (2017). Individual fibre segmentation from 3d x-ray computed tomography for characterising the fibre orientation in unidirectional composite materials. Composites Part A: Applied Science and Manufacturing.

[38] Emerson MJ, Dahl VA, Conradsen K, Mikkelsen LP, Dahl AB (2018). Statistical validation of individual fibre segmentation from tomograms and microscopy. Composites Science and Technology.

[39] Creveling PJ, Whitacre WW, Czabaj MW (2019). A fiber-segmentation algorithm for composites imaged using x-ray microtomography: Development and validation. Composites Part A: Applied Science and Manufacturing.

[40] Yu, H. *et al*. Unsupervised learning for large-scale fiber detection and tracking in microscopic material images. *arXiv:1805.10256 [cs]*. ArXiv: 1805.10256 (2018).

[41] Ren S, He K, Girshick R, Sun J (2017). Faster r-cnn: Towards real-time object detection with region proposal networks. IEEE transactions on pattern analysis and machine intelligence.

[42] Miramontes, S., D. M.D. Y.*et al*. (eds.) *Advances in Visual Computing*, Lecture Notes in Computer Science, 541–552, 10.1007/978-3-030-33723-0_44 (Springer International Publishing, 2019).

[43] Lecun Y, Bottou L, Bengio Y, Haffner P (1998). Gradient-based learning applied to document recognition. Proceedings of the IEEE.

[44] Abadi, M. *et al*. Tensorflow: a system for large-scale machine learning. In *Proceedings of the 12th USENIX conference on Operating Systems Design and Implementation*, OSDI’16, 265–283 (USENIX Association, 2016).

[45] Chollet, F. *et al*. Keras. https://keras.io (2015).

[46] Srivastava N, Hinton G, Krizhevsky A, Sutskever I, Salakhutdinov R (2014). Dropout: a simple way to prevent neural networks from overfitting. The Journal of Machine Learning Research.

[47] Kingma, D. P. & Ba, J. Adam: A method for stochastic optimization. *arXiv:1412.6980 [cs]*. ArXiv: 1412.6980 (2017).

[48] Dauphin, Y. N., de Vries, H. & Bengio, Y. Equilibrated adaptive learning rates for non-convex optimization. *arXiv:1502.04390 [cs]*. ArXiv: 1502.04390 (2015).

[49] Ioffe, S. & Szegedy, C. Batch normalization: Accelerating deep network training by reducing internal covariate shift. *arXiv:1502.03167 [cs]*. ArXiv: 1502.03167 (2015).

[50] Hunter JD (2007). Matplotlib: A 2d graphics environment. Computing in Science & Engineering.

[51] Yoo TS (2002). Engineering and algorithm design for an image processing api: A technical report on itk - the insight toolkit. Studies in health technology and informatics.

[52] Kluyver, T. *et al*. Jupyter notebooks – a publishing format for reproducible computational workflows. In Loizides, F. & Schmidt, B. (eds.) *Positioning and Power in Academic Publishing: Players, Agents and Agendas*, 87–90 (IOS Press, 2016).

[53] Fioravante de Siqueira A, Van Der Walt S, Ushizima DM (2021). Dryad.

